# Editorial Comment to Significance of dorsal bladder neck involvement in predicting the progression of non‐muscle‐invasive bladder cancer

**DOI:** 10.1111/iju.15150

**Published:** 2023-01-18

**Authors:** Angelo Naselli, Giacomo Maria Pirola

**Affiliations:** ^1^ Urology Department San Giuseppe Hospital, IRCCS Multimedica, Multimedica Group Milan Italy

In this relevant paper, the authors outline, for the first time, the impact of dorsal bladder neck involvement (BNI) on the progression rate of non‐muscle‐invasive bladder cancer (NMIBC).^1^ To demonstrate this statement, the authors report their wide retrospective single‐center casistic (651 NMIBC patients over 18 years), excluding cases with concomitant upper urinary tract cancer, carcinoma in‐situ or de novo metastatic disease. A simple division of anatomical location of first‐case presentation (dorsal BNI, ventral BNI and non‐BNI) showed a significantly increased rate of large diameter tumor (>3 cm), positive cytology, secondary resection (re‐TURBT) and need of bladder BCG instillations in the dBNI group compared to the others. Moreover, also the progression rate was higher in cases of first dBNI presentation (16% vs. 4.9% for cases without BNI) with an estimated 5 years progression rate of 12% versus 5%. Therefore, the authors found in multivariate analysis that dBNI was an independent risk factor for disease progression (HR 2.89, *p* 0.025) as well as tumor stage pT1 (HR 4.02 *p* 0.001) and histological grade G3 (HR 3.33, *p* 0.003). Obviously, those data need an external validation. In the meanwhile, we may hypothesize the etiology. NMIBC recurrence and progression rate can be reduced by means of a correct endoscopic resection (TURBT) and tailored surveillance or bladder instillations. Therefore, beyond factors suggested by the authors, TURBT at the dBNI may be more challenging for various anatomical factors like the proximity of ureteral orifices or a particular acute angulation between the middle lobe and the trigone in the male. Consequently, sometimes, the resection may be incomplete and/or the specimen may be inadequate impairing cancer control.^2^ Therefore, the relevance of dBNI on disease progression should be evaluated when facing this kind of tumors. Future trials will clarify if a secondary TURBT or a subsequent cycle of BCG instillation can reduce the risk of progression, or if a closer follow‐up can also be considered a safe approach. Even with the intrinsic limitations of a single‐center casistic, the authors should be congratulated for this new finding as it has the potential to be recognized as a disease site to be further investigated similar to prostatic urethra. We are sure that further articles on this subject will help to have a wider overview on this presentation of NMIBC.

## CONFLICT OF INTEREST

No conflict of interests has to be disclosed.
